# Trends of Soil and Solution Nutrient Sensing for Open Field and Hydroponic Cultivation in Facilitated Smart Agriculture

**DOI:** 10.3390/s25020453

**Published:** 2025-01-14

**Authors:** Md Nasim Reza, Kyu-Ho Lee, Md Rejaul Karim, Md Asrakul Haque, Emmanuel Bicamumakuba, Pabel Kanti Dey, Young Yoon Jang, Sun-Ok Chung

**Affiliations:** 1 Department of Agricultural Machinery Engineering, Graduate School, Chungnam National University, Daejeon 34134, Republic of Korea; reza5575@cnu.ac.kr (M.N.R.); elkyu0927@cnu.ac.kr (K.-H.L.); mrkarim@o.cnu.ac.kr (M.R.K.); asrak.bzs13@o.cnu.ac.kr (M.A.H.); ebicamu@o.cnu.ac.kr (E.B.); pabel@o.cnu.ac.kr (P.K.D.); 2 Department of Smart Agricultural Systems, Graduate School, Chungnam National University, Daejeon 34134, Republic of Korea; 3 SUNGBOO Ind. Co., Ltd., Chilgok 39909, Republic of Korea; chang1y@hanmail.net

**Keywords:** precision agriculture, soil nutrient, hydroponic solution, nutrient sensing, sensing technologies, open field

## Abstract

Efficient management of soil nutrients is essential for optimizing crop production, ensuring sustainable agricultural practices, and addressing the challenges posed by population growth and environmental degradation. Smart agriculture, using advanced technologies, plays an important role in achieving these goals by enabling real-time monitoring and precision management of nutrients. In open-field soil cultivation, spatial variability in soil properties demands site-specific nutrient management and integration with variable-rate technology (VRT) to optimize fertilizer application, reduce nutrient losses, and enhance crop yields. Hydroponic solution cultivation, on the other hand, requires precise monitoring and control of nutrient solutions to maintain optimal conditions for plant growth, ensuring efficient use of water and fertilizers. This review aims to explore recent trends in soil and solution nutrient sensing technologies for open-field soil and facilitated hydroponic cultivation, highlighting advancements that promote efficiency and sustainability. Key technologies include electrochemical and optical sensors, Internet of Things (IoT)-enabled monitoring, and the integration of machine learning (ML) and artificial intelligence (AI) for predictive modeling. Blockchain technology is also emerging as a tool to enhance transparency and traceability in nutrient management, promoting compliance with environmental standards and sustainable practices. In open-field soil cultivation, real-time sensing technologies support targeted nutrient application by accounting for spatial variability, minimizing environmental risks such as runoff and eutrophication. In hydroponic solution cultivation, precise solution sensing ensures nutrient balance, optimizing plant health and productivity. By advancing these technologies, smart agriculture can achieve sustainable crop production, improved resource efficiency, and environmental protection, fostering a resilient food system.

## 1. Introduction

Agriculture has always been the backbone of human civilization, and with the global population expected to exceed 9 billion by 2050, the demand for sustainable food production is increasing rapidly [1,2]. To meet these growing food needs, the agricultural sector has undergone a transformation driven by technological advancements, and among these innovations, smart agriculture plays an important role [3]. Smart agriculture incorporates advanced technologies such as Internet of Things (IoT), artificial intelligence (AI), and machine learning (ML) to enhance productivity and sustainability in farming [4,5]. One of the key areas where these technologies are making a significant impact is in soil nutrient sensing, which is essential for both open field soil cultivation and facilitated hydroponic solution cultivation systems [3].

Soil is the most important and sensitive resource for agricultural crop production, essential for maintaining ecosystem balance and ensuring sustainable food production [6,7]. To enhance soil fertility and maintain the nutritional balance, chemical fertilizers and organic matter are commonly applied. However, in many cases, chemical fertilizers are used in excessive and disproportionate quantities to increase crop yields under conditions of nutrient imbalance [8,9]. This overuse of fertilizers can lead to the deterioration of both soil health and the surrounding environment. Low soil fertility often results from increased crop productivity without the proper restoration of essential nutrients. Intensive farming practices, while aimed at maximizing output, can degrade soil quality over time, necessitating the balanced and precise application of fertilizers to restore essential nutrients and maintain soil health [10].

Nutrients are fundamental to healthy plant growth and are crucial for enhancing agricultural productivity [11]. Plants depend on a range of essential nutrients, including macronutrients like nitrogen (N), phosphorus (P), and potassium (K), as well as secondary nutrients such as calcium (Ca), magnesium (Mg), and sulfur (S), to support key biological functions [10,12,13,14]. Nitrogen drives chlorophyll production and photosynthesis, phosphorus is critical for energy transfer and root development, while potassium regulates water balance and enzyme activation [15]. Additionally, micronutrients, including iron (Fe), manganese (Mn), zinc (Zn), and copper (Cu), are vital for specific physiological functions that contribute to robust growth, disease resistance, and high yields [16]. Nutrient deficiencies significantly affect plant growth and productivity. While much research has focused on individual nutrient shortages (e.g., N, P, S, Fe, Zn), there is growing recognition of the complex molecular interactions between nutrients [17,18]. Macronutrients such as N, P, and S not only interact with each other but also influence the metabolic pathways of key micronutrients [12,17]. Understanding these intricate relationships is essential for optimizing nutrient management strategies, ensuring a balanced nutrient supply, and promoting sustainable agricultural practices [16].

Soil nutrients, including essential elements like nitrogen (N), phosphorus (P), and potassium (K), are fundamental to plant growth, and their effective management directly impacts crop yield and quality [17,19,20]. Neocleous and Savvas [21] found that maintaining nutrient levels within ±10% of target concentrations increased tomato yield by 25% compared to standard practices. Nutrient imbalances can have significant negative effects: excess nitrogen consumption, reducing fruit quality and increasing pest susceptibility [22]. Short-term deficiencies can also cause long-lasting impacts. Signore et al. [23] reported that a 48-h iron deficiency in hydroponic spinach led to a 40% reduction in final yield, even after the deficiency was corrected. Monitoring and sensing soil nutrients is a need for maintaining optimal nutrient levels, which directly enhance crop yield and quality. Traditionally, farmers relied on periodic manual soil testing to assess nutrient levels and make fertilization decisions. However, these methods are often labor-intensive, time-consuming, and do not provide real-time insights [24,25]. As a result, there is a growing need for more efficient, real-time soil nutrient sensing technologies that allow farmers to monitor and manage nutrient levels more precisely. This shift is particularly important in both open field soil cultivation and controlled environments like hydroponic solution systems, where nutrient availability must be closely monitored to ensure optimal plant growth [26,27].

Recent advancements in soil nutrient sensing technologies aim to address these challenges by offering precise, real-time insights into nutrient levels. For open field soil cultivation, a range of sensors such as electrochemical, optical, and remote sensing platforms have been developed [28,29]. These technologies allow for the detection of specific nutrient concentrations and spatial variations in nutrient distribution across large fields, enabling farmers to practice precision agriculture [30]. Soil nutrient sensing supports site-specific nutrient management (SSNM) by recognizing varying nutrient needs within a field [31,32]. This technology enables differential fertilizer application based on real-time data, reducing the risks of over-fertilization, which can cause eutrophication, and under-fertilization, which can lower yields [32,33]. SSNM improves nutrient efficiency, ensuring crops receive the precise nutrients needed for optimal growth. A key element of SSNM is variable-rate technology (VRT), which allows dynamic application of fertilizers, herbicides, and water [31,34]. Integrated with soil nutrient sensing, VRT makes real-time adjustments across field zones, minimizing nutrient imbalances, maximizing yields, and lowering input costs [31,32]. This data-driven approach ensures that fertilizers are applied for the open fields only where and when needed, optimizing resource use and minimizing waste [34].

In agriculture, a variety of soil sensing principles and sensor mounting platforms are used to monitor soil health and optimize nutrient management [35]. Key sensing technologies include electrochemical sensors, which measure specific ions like N, P, K and S by detecting electrical conductivity or potential changes [35,36]. Optical sensors, using near-infrared (NIR) or mid-infrared (MIR) spectroscopy, provide non-invasive analysis of soil properties such as organic matter, moisture, and nutrient content [37]. Capacitive sensors measure soil moisture, critical for efficient irrigation management, while thermal and electrical sensors offer insights into soil temperature and structure [38]. These sensors are mounted on platforms such as handheld devices [39] for spot-checking, drones equipped with optical or multispectral sensors [40] for large-area mapping, and tractors or autonomous vehicles [41,42,43] for continuous, real-time data collection as they move across fields. Together, these technologies enable precise monitoring and management of soil conditions, improving nutrient use efficiency, crop yields, and overall agricultural sustainability [40,42].

Hydroponic solution cultivation systems are becoming popular in protected horticulture due to their ability to enhance plant growth and yield with minimal maintenance costs [44]. In these systems, the nutrient solution directly supports plant growth by allowing exposed roots to absorb nutrients, reducing the need for weed control and minimizing the use of chemicals and pesticides [45,46,47]. Additionally, nutrient solution sprays help sterilize roots, preventing diseases, while maximizing root surface contact with nutrients [48]. Sensor-based aeroponic systems with recycling capabilities are gaining attention for their efficient water and nutrient use, reduced environmental impact, sustainability, and cost-effectiveness [49,50,51]. By enabling real-time monitoring and adjustment of nutrient levels, it ensures efficient fertilizer and water use, while maximizing crop yield and quality, which is crucial for the commercialization of horticultural products. In hydroponic solution cultivation, sensors such as pH meters, electrical conductivity (EC) sensors, and ion-selective electrodes (ISE) are used to continuously monitor nutrient levels in water solutions [44,52]. These sensors are often integrated with automated dosing systems that adjust nutrient concentrations in real-time, ensuring plants receive a balanced supply of nutrients for optimal growth [53].

Furthermore, the convergence of multiple sensor types into integrated platforms is driving the future of nutrient sensing. These multi-sensor systems combine data from various sources—such as pH, EC, and ion-specific sensors—to provide a comprehensive understanding of soil or nutrient solution conditions, enabling farmers to make more accurate and holistic decisions. The potential benefits of smart nutrient sensing technologies are profound, particularly when integrated with machine learning and AI systems, which allow for predictive modeling and more informed decision-making [54]. In open field soil farming, sensing technologies can help mitigate the effects of environmental variability, while in hydroponic solution cultivation enable more precise control of nutrient solutions, leading to higher yields and resource efficiency [31,32,36,37]. Additionally, the miniaturization and cost reduction of sensors have made these technologies more accessible, encouraging wider adoption among farmers, including those in resource-limited regions [55]. Blockchain technology is also being explored to improve transparency and traceability in nutrient management, enabling farmers to verify sustainable practices and meet environmental standards [56].

As agriculture continues to evolve, soil nutrient sensing technologies will play an increasingly important role in supporting global food security and environmental sustainability. By providing real-time, data-driven insights into nutrient dynamics, these technologies allow farmers to optimize fertilization practices, improve crop yields, reduce input costs, and minimize environmental impact. This makes them essential tools in the shift towards more sustainable and resilient farming systems.

Advancements in soil nutrient sensing technologies have been remarkable, yet a significant gap persists in bridging traditional open-field cultivation and modern hydroponic systems. Current research often focuses exclusively on one method, missing opportunities to explore synergies and cross-applicability. For instance, while hydroponic ion-selective electrodes excel in controlled environments, their potential for open-field site-specific management remains underexplored. Similarly, technologies like electrochemical sensors, proven in open-field applications, have not been fully assessed for hydroponic systems, where nutrient dynamics differ. A comparative analysis is required to evaluate the adaptability and performance of these technologies across diverse cultivation systems. Another critical gap lies in the inconsistent evaluation of sensor effectiveness. Most studies lack standardized protocols to assess reliability, accuracy, and cost-effectiveness under varying conditions. For example, while ion-selective electrodes perform well in hydroponic setups, their sensitivity and durability can degrade in open-field soils with fluctuating pH, texture, and salinity. Without a unified framework for evaluating performance metrics, such as response time, calibration needs, and cost-efficiency, it becomes difficult to compare technologies or adapt them effectively across systems.

Besides, practical and technological barriers significantly limit the broader adoption of nutrient sensing technologies. Challenges such as insufficient sensitivity to trace elements, environmental impacts on sensor accuracy, and durability in heterogeneous soils remain unresolved. Additionally, high costs, complex maintenance, and limited scalability hinder accessibility, especially for small-scale farmers. Addressing these issues requires the development of robust, affordable sensors with improved sensitivity, automated calibration, and scalable designs.

These gaps highlight the necessity of a literature review that bridges knowledge across cultivation systems and offers insights into both technical and practical aspects of nutrient sensing technologies. While there has been significant advancement in soil nutrient sensing technologies, there is a lack of comprehensive analysis [16,31,32,39] of their applications in both traditional open-field cultivation and modern hydroponic systems. Most existing reviews [42,48,50,52] focus exclusively on one of these domains, limiting a broader understanding of cross-applicability and synergies. Current studies [19,21,41,44] often lack standardization in evaluating the performance, reliability, and cost-effectiveness of various nutrient sensors across diverse agricultural systems.

Variations in environmental conditions, crop types, and system-specific requirements remain underexplored, leading to fragmented knowledge. There is insufficient synthesis [19,20,31,49] of how technological limitations, such as sensitivity, accuracy, and real-time usability, and practical barriers, like cost and scalability, impact the broader implementation of these technologies in sustainable farming practices. Despite advancements in sensor designs, high costs and technical complexities often restrict access to smallholder farmers, limiting the scalability of these technologies.

The objective of this article was to deliver a comprehensive literature review on soil and solution nutrient sensing technologies tailored for open-field and facilitated smart agriculture systems. This article highlights the important role of nutrient sensing technologies in advancing agricultural productivity and sustainability across open-field and hydroponic systems. It examines advancements in electrochemical, optical, and multi-sensor platforms for precise monitoring of soil and solution nutrient levels. The integration of IoT, machine learning (ML), artificial intelligence (AI), and blockchain technologies is explored for their potential to enable real-time decision-making, predictive modeling, and efficient nutrient management. Benefits of hydroponic systems are emphasized, showcasing automated nutrient management through pH meters, EC sensors, and ion-selective electrodes (ISE). Challenges such as affordability, accessibility, and technical limitations are addressed, with a focus on innovations like sensor miniaturization and cost-effective solutions to enhance adoption in resource-limited regions.

## 2. Method

The primary objective of this study was to systematically examine, summarize, and synthesize the vast body of existing research, technological advancements, and practical applications in soil nutrient sensing technologies, specifically for open-field and hydroponic cultivation systems. This review aimed to provide a holistic and critical perspective on state-of-the-art innovations, bridging the knowledge gap between traditional and modern cultivation methodologies. By analyzing literature published from 2010 to 2024, the review captures the rapid evolution of sensing technologies in precision agriculture and their essential role in sustainable farming practices.

The review process was accurately carried out by sourcing peer-reviewed articles, technical reports, and conference proceedings from prominent academic databases, including Google Scholar, Web of Science, IEEE Xplore, Elsevier and ScienceDirect. These databases were selected for their extensive coverage of multidisciplinary topics, encompassing agronomy, engineering, environmental science, and data analytics. Keywords such as “soil nutrient sensing”, “precision agriculture”, “hydroponic nutrient management”, “sensor technology in agriculture”, “hydroponic solution”, “open field”, and “sustainable farming practices” were used to ensure a comprehensive collection of relevant studies. The inclusion criteria focused on articles with significant contributions to sensor technologies, including advancements in electrochemical, optical, and remote sensing techniques, as well as applications in decision support systems (DSS) and Internet of Things (IoT) platforms.

For this review, 156 research papers, technical reports, and review articles were selected from a comprehensive pool of literature published between 2010 and 2024. The collected documents were systematically categorized based on the core sub-sections as shown in Table 1. To analyze and present the data, the review employed a thematic analysis framework, guided by established methodologies [57,58]. Thematic categorization identified key trends such as soil nutrient sensing, hydroponic nutrient management, precision agriculture, sensor technologies in agriculture, IoT integration, AI-driven nutrient modeling, and field applications, grouping studies under themes like technological innovations, cultivation system applications, and deployment challenges. A comparative analysis evaluated sensor performance metrics (e.g., accuracy, sensitivity, cost) across open-field and hydroponic systems, emphasizing differences in nutrient sensing adaptability. The conceptual framework assessed the role of sensing technologies in precision agriculture practices, such as site-specific nutrient management (SSNM) and variable-rate fertilization (VRF), aligning data with sustainability goals like resource efficiency. Finally, the synthesis highlighted challenges, such as sensor standardization and integration barriers, and proposed innovative solutions, including hybrid sensors and machine-learning models, addressing gaps in current research. This structured approach ensured a comprehensive and critical evaluation of nutrient sensing technologies for sustainable agriculture.

The choice of performing a literature review rather than a systematic review for this manuscript is grounded in the nature of the research objectives and the breadth of the topic under investigation. A literature review provides the flexibility required to explore the diverse and interdisciplinary advancements in soil and hydroponic nutrient sensing technologies, which span fields such as agronomy, sensor technology, data science, and environmental engineering. This approach enables the synthesis of key trends, emerging innovations, and practical applications, which may not align with the rigid, protocol-driven methodology of a systematic review. A systematic review is designed to answer highly specific research questions by employing a structured and reproducible method for selecting, analyzing, and synthesizing studies [57]. While this approach ensures comprehensiveness and minimizes bias, it may not capture the exploratory nature required to address the broader research gaps highlighted in this manuscript. Additionally, as Hart [58] emphasizes, a literature review is ideal for establishing a conceptual framework and identifying patterns and relationships in existing research. This approach supports the goal of providing a comprehensive overview of nutrient sensing technologies, summarizing their advancements, and identifying future research directions. Unlike the narrower scope of a systematic review, a literature review allows for the inclusion of diverse perspectives and emerging topics, which are critical for an evolving field like precision agriculture.

## 3. Results

This section presents a comprehensive synthesis of the findings from the reviewed literature, focusing on advancements, challenges, and applications in nutrient sensing technologies for agriculture. The results are organized to provide a clear progression of topics, beginning with the identification of key soil sensing parameters essential for monitoring soil health and nutrient status. This is followed by a detailed analysis of sensing techniques, including electrochemical, optical, and remote sensing methods, emphasizing their roles in improving nutrient management.

The section further explores into soil nutrient sensing for site-specific open-field management, highlighting the dynamics of soil nutrients, the deployment of advanced sensing platforms, and their integration with decision support systems to enhance precision agriculture practices. Lastly, it explores solution nutrient sensing for facilitated hydroponic horticulture, focusing on nutrient management strategies, innovative sensing technologies, and the role of automation and real-time monitoring in optimizing resource use and crop productivity. Together, these findings provide actionable insights into the potential of nutrient sensing technologies to advance sustainable agricultural practices.

### 3.1. Nutrient Sensing

#### 3.1.1. Key Soil Sensing Parameters

**Figure 1:**
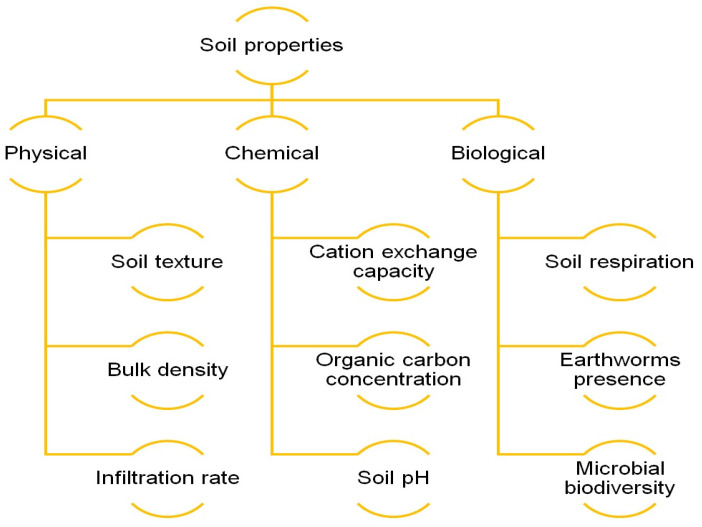
The classification of soil properties into physical, chemical, organic carbon concentration, and biological parameters (Synthesized using comparative analysis of soil properties and parameters from [59]). These properties collectively influence soil behavior, nutrient cycling, water retention, and the capacity to support plant growth and ecosystem functions.

Soil nutrient sensing measures key parameters influencing soil health, productivity, and plant growth, offering vital insights into nutrient status and fertility. Soil nutrient sensing parameters are broadly categorized into physical, chemical, and biological parameters which are essential for soil-plant interactions and ecosystem functioning [59]. Figure 1 illustrates the fundamental physical, chemical, and biological properties commonly assessed to evaluate soil properties.

The physical properties of soil are fundamental to water retention, aeration, and root development. Soil texture, defined by the relative proportions of sand, silt, and clay, affects water-holding capacity and drainage [60]. Bulk density and soil compaction influence root penetration and regulate air and water movement. Soil moisture, essential for plant water availability, closely correlates with electrical conductivity (EC) [31,61]. Additionally, soil temperature and relative humidity (RH) also significantly influence seed germination, root development, and microbial activity [62].

Chemical properties are essential for plant nutrition and fertility. Macronutrients like nitrogen (N), phosphorus (P), and potassium (K) support growth, while micronutrients, though needed in smaller amounts, are vital for physiological functions [20]. Soil pH affects nutrient availability, with optimal uptake in a slightly acidic to neutral range. Salinity, measured via EC, must be managed to prevent reduced crop yields and plant stress. Biological properties indicate soil health and ecosystem function [63]. Organic matter improves structure, water retention, and microbial activity. Cation exchange capacity (CEC) reflects the ability to retain and release nutrients, ensuring availability to plants [64]. Soil respiration, measuring microbial decomposition of organic matter, serves as a key indicator of microbial activity and overall soil health [62].

Soil sensing enables the measurement and monitoring of key physical and biochemical attributes, such as nutrients and water, to better understand their dynamics, interactions, and spatial variability within the environment. Schmidinger et al. [65] identified six essential soil parameters to monitor: soil organic carbon, pH, moisture, and plant-available phosphorus, magnesium, and potassium. Different soil sensing techniques can be applied to measure a wide range of parameters, including soil moisture, nutrients, cation exchange capacity (CEC), organic carbon, pH, soil texture (clay, silt, and sand), mineralogy, soil strength, and bulk density [66].

#### 3.1.2. Soil Nutrient Sensing Technique

Soil nutrient sensing techniques refer to various methods used to assess the availability and concentration of essential nutrients in soil [54,55]. These techniques provide essential information for farmers, environmental scientists, and policymakers to optimize fertilizer use, manage crop yields, and ensure sustainable agricultural practices [32,33,42]. The classification of these techniques can be based on different factors, such as the type of technology, data collection method, application environment, or the frequency of measurement. Traditional soil nutrient sensing technology for acquiring nutrient information is costly, time-consuming, and poor in real time and is not suitable to large-scale farmland [25]. Thus, it is necessary to detect the status of nutrient parameters rapidly and accurately in large-scale areas [43].

**Figure 2:**
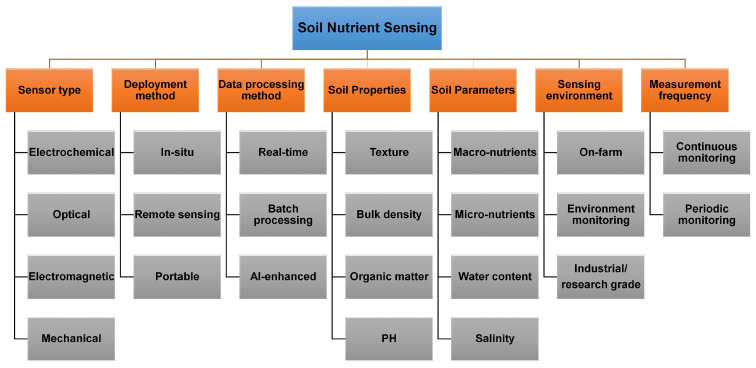
Overview of soil nutrient sensing techniques for nutrient management (Synthesized using comparative analysis of soil properties, parameters, and sensing techniques from [40,59,67]).

To fully realize the potential of soil nutrient in delivering multiple functions, a comprehensive understanding of its behavior and variability across spatial and temporal scales is essential [32]. However, direct measurement of these soil functions poses significant challenges. Sensing technologies, including laboratory spectroscopy, ground-based, proximal, and remote sensing, offer reliable means to assess soil variability at various scales, facilitating the study of soil functionality [31,32,67]. Laboratory spectroscopy, recognized as a global tool, accurately characterizes a wide range of soil physical, chemical, and biological properties [68]. Proximal sensing, utilizing diverse sensors, provides practical solutions for in-field soil characterization [66]. Remote sensing has proven effective for monitoring soil conditions over large spatial and temporal extents [40,67]. These sensing technologies enable a more comprehensive assessment of soil functions across local, regional, and global levels, advancing research and sustainable soil management practices [67]. Figure 2 shows different types of soil nutrient sensing techniques based on different factors, such as the type of technology, data collection method, application environment, or the frequency of measurement.

Soil nutrient sensing techniques can be categorized based on the types of sensors used, each offering unique methods to assess soil quality and nutrient availability. Electrochemical sensors measure soil nutrients by monitoring ion concentrations such as pH, nitrate, or potassium levels, with ion-selective electrodes (ISEs) and ion-sensitive field-effect transistors (ISFETs) being common examples [46,66]. These sensors provide real-time data but require regular calibration and maintenance. Optical sensors use spectroscopy, including visible, near-infrared (NIR), and mid-infrared (MIR) wavelengths, to detect chemical signatures related to soil nutrients [68,69]. Non-invasive and quick, they employ technologies such as diffuse reflectance spectroscopy and laser-induced breakdown spectroscopy (LIBS) to offer precise nutrient assessments [47,68]. Electromagnetic sensors, like Ground Penetrating Radar (GPR) and Electromagnetic Induction (EMI), assess soil moisture and conductivity, indirectly reflecting nutrient availability [48,70]. The mechanical sensors assess soil compaction and related physical parameters influencing nutrient uptake. While tools like penetrometers provide indirect information about nutrient availability, they play a critical role in determining soil structure and health [71]. These sensors work together to enable targeted interventions, enhancing precision agriculture for better crop yields and soil management.

Soil nutrient sensing techniques can also be classified by their deployment method or platform. In-situ sensors are embedded directly into the soil, providing continuous real-time monitoring to support precision agriculture by optimizing fertilizer use, though they may degrade over time due to environmental exposure [72]. Remote sensing techniques rely on sensors mounted on satellites, drones, or other airborne platforms to monitor large areas [50,67]. Using multispectral and hyperspectral imaging, these techniques detect nutrient deficiencies by analyzing changes in vegetation health, color, and reflectance [73]. Portable sensors offer on-site nutrient measurements, with handheld devices like portable spectrometers and electrochemical sensors enabling farmers and agronomists to conduct quick, localized assessments in the field [55,74].

Soil nutrient sensing techniques can also be classified by data collection and processing methods. Real-time sensors provide immediate feedback, enabling quick decision-making, with electrochemical sensors like nitrate probes commonly used in precision agriculture [24,27,29,72]. Batch processing sensors involve collecting soil samples for later laboratory analysis using methods like spectroscopy, atomic absorption spectrometry (AAS), or chemical assays, offering high accuracy but lacking real-time insights [47,68,75]. AI- and machine learning-enhanced sensors integrate data from various sources—such as optical, electrochemical, and environmental sensors—to deliver comprehensive soil health assessments and predict nutrient deficiencies [4,54].

Based on measurement frequency, continuous monitoring systems collect real-time data via wireless sensor networks (WSNs), supporting automated agricultural systems with dynamic decision-making [76,77]. In contrast, periodic or seasonal monitoring systems perform scheduled measurements, such as weekly or seasonal assessments, with satellite-based sensors commonly used to track crop development and soil nutrient levels over time [29,39]. Classified by analytical approach as direct or indirect measurement sensors, like electrochemical probes, measure specific nutrients such as nitrates or phosphates directly in the soil [32,66]. Indirect measurement techniques estimate nutrient availability through correlated parameters, such as soil conductivity, organic matter, or plant health, with remote sensing methods inferring nutrient status by analyzing vegetation patterns [49,67].

Non-contact soil nutrient sensing techniques measure properties without direct contact, with proximal sensing used within 2 m of the target and remote sensing for greater distances. Rossel et al. [66] define proximal sensing as field-based sensing, either in contact with or near the soil. It includes both invasive and non-invasive methods, utilizing active or passive energy sources. The main advantage against invasive techniques is the possibility of reading from different locations within a reasonable timeframe and naturally, not to touch nor affect the process [26,44]. This ability to move the sensors and instruments allows overcoming the disadvantage of the invasive types with the small volume of the sample being measured [28,34]. Non-invasive sensing is also easier to deploy and result in more stable results in harsh environments such as rocky or vertical soil, not depending on surface contacts.

#### 3.1.3. Importance of Soil Nutrient Parameter

Water is essential for life processes, with no life cycle possible without it. In plants, nutrient uptake through roots is facilitated by soil water, making both water and soil fundamental to plant growth and development [78]. Soil water content, indicates the amount of water present in soil and is defined as the ratio of water to solid particles in a soil sample [79]. Soil water content plays an important role in shaping the engineering, agronomic, ecological, biological, and hydrological properties of soil, particularly in open-field agriculture and indoor horticultural crop production [49,64].

Soil consists of a three-phase system: solid minerals, water, and air, with water content influencing key mechanical properties such as consistency, compaction, cracking, swelling, shrinkage, and density [64,80]. Soil water exists in three forms: gravitational, capillary, and hygroscopic moisture [81]. Gravitational water moves freely under the influence of gravity and is unavailable to plants, while capillary water resides in pores, driving interactions between soil, nutrients, and the environment [82]. Hygroscopic water forms a thin film around soil particles, contributing to soil-plant interactions. Soil water content affects the transition between dry and saturated states, impacting plant growth according to environmental conditions [82]. Accurate measurement of soil moisture is therefore crucial in agriculture, as soil physical, chemical, mineralogical, mechanical, geotechnical, hydrological, and biological properties depend heavily on moisture levels [24,27]. Understanding soil water dynamics is essential for optimizing crop production and soil management practices [45,48].

**Figure 3:**
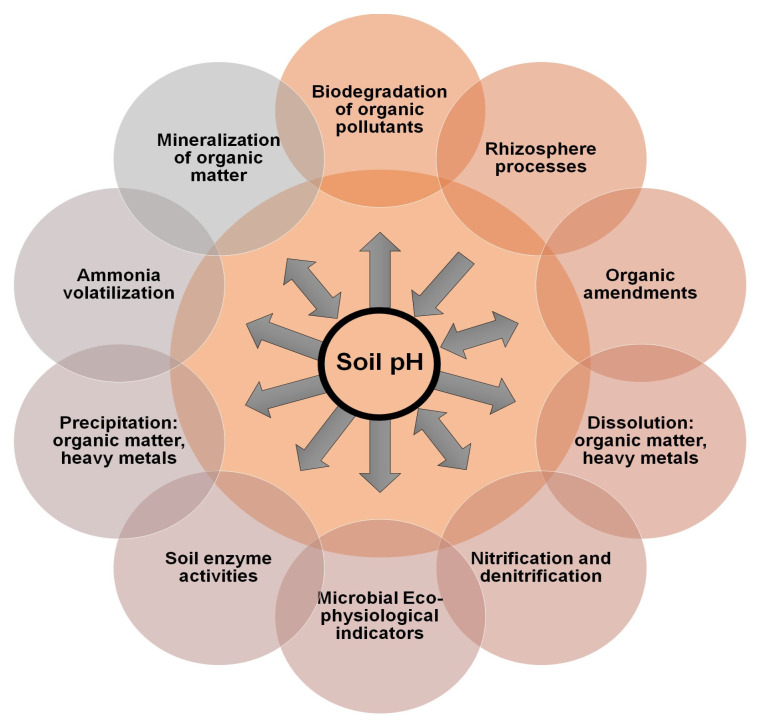
The influence of soil pH on key biogeochemical processes (modified from Neina [85]).

It is well-established that 17 essential nutrients are critical for plant growth, and the deficiency of any one can lead to yield reduction [83,84]. Carbon (C), hydrogen (H), and oxygen (O) are readily available from the atmosphere, while mineral elements like nitrogen (N), phosphorus (P), and potassium (K), known as primary macronutrients, are required in large quantities and must be supplemented through fertilizers to achieve optimal crop yields. Secondary macronutrients, including calcium (Ca), magnesium (Mg), and sulfur (S), are needed in smaller amounts and are typically present in sufficient quantities within the soil [83]. Soil pH, often referred to as the “master soil parameter”, significantly affects biological, chemical, and physical properties of soil, influencing both plant growth and biomass yield [85]. It plays a role in regulating biochemical processes within the soil environment, directly impacting nutrient availability and overall soil health [85,86]. Numerous biogeochemical processes essential for maintaining soil functionality are pH dependent, as illustrated in Figure 3. Additionally, soil moisture is crucial for promoting microbial diversity, which supports plant growth by fostering beneficial soil-plant interactions [87].

Soil temperature is a key factor influencing soil moisture, plant growth, vegetation, and soil formation. It affects microbial activities, abiotic chemical processes, and biochemical reactions within the soil [88]. Studies highlight soil temperature as a critical regulator, particularly in agriculture, organic waste treatment, and biological systems related to plant growth [89]. Physical, chemical, and microbiological processes are temperature-dependent, influencing contaminant transport and behavior in subsurface environments [90]. Additionally, soil temperature plays a vital role in seed germination, crop production, soil respiration, and the proliferation of soil-borne pests [91].

Soil salinity is a parameter characterized by the accumulation of excess ions such as calcium (Ca^2+^), magnesium (Mg^2+^), sodium (Na^+^), sulfates (SO_4_^2−^), and chlorides (Cl^−^) in the soil [92]. It inhibits plant growth and physiological functions, disrupts nutrient and water uptake, and poses a major threat to soil health, contributing significantly to land degradation. Shrivastava et al. [93] identified salinity as a key environmental factor limiting crop productivity, as most crops are highly sensitive to salt stress. Salinity issues are worsening with the continuous expansion of salt-affected land, resulting in substantial yield losses, ranging between 20% and 50%, particularly when combined with drought conditions [94].

Soil cation exchange capacity (CEC) measures the ability to retain exchangeable cations, reflecting the negative charge per unit mass of soil [93]. This property is essential for maintaining nutrient availability, as it determines the capacity to hold essential ions like calcium, magnesium, and potassium. Soil consists of both mineral and organic components, exhibiting chemical, physical, mineralogical, and biological characteristics that collectively support plant growth and play a crucial role in agricultural productivity [94]. Soil mineralogy focuses on the composition and structure of minerals, which significantly influence the physical properties and fertility [95]. The breakdown of primary minerals and the formation of secondary minerals affect soil texture, structure, and its ability to retain nutrients and water. The mineral composition is closely linked to the chemical properties, shaping the capacity to support plant growth and adapt to environmental changes [96]. Understanding these relationships is critical for effective soil management and sustainable agricultural practices.

### 3.2. Soil Nutrient Sensing for Site-Specific Open Field Management

Site-specific nutrient Management is a precision agriculture approach that focuses on applying the precise amount of nutrients to crops based on their specific location and growth stage, instead of using the same fertilizer recommendations for the entire field [97]. Open fields, characterized by varying soil conditions, nutrient dynamics, and environmental factors, require site-specific nutrient management strategies to optimize crop yield and minimize environmental impact [98]. This section explores how sensor technologies are employed to monitor and assess soil nutrient levels, ensuring precise input of fertilizers and amendments where they are needed the most. Integrating these technologies into broader decision support systems further enhances data-driven approaches to field management.

#### 3.2.1. Soil Nutrient Dynamics in Open Fields

Soil nutrient dynamics in open fields are governed by complex interactions between biological, chemical, and physical processes that determine the availability and mobility of essential nutrients for plant growth. Understanding these dynamics is critical for implementing precise, site-specific nutrient management practices that optimize crop yield while minimizing environmental impacts. In open-field environments, the spatial and temporal variability of soil nutrients presents unique challenges for effective monitoring and management. Nutrient dynamics in soil are influenced by several variables, including soil texture [99], organic matter content [100], pH levels [101], water availability [102], and crop type [103]. Nutrient sensing techniques must consider the variation in soil conditions, providing useful data that can help guide site-specific management strategies [104]. The following sections provide a detailed examination of the factors influencing nutrient availability, the spatial variability of nutrients, and the implications of these dynamics for nutrient sensing and management strategies.

Nutrient availability in open-field soils is shaped by a combination of soil texture, organic matter content, pH, moisture levels, microbial activity, and the presence of other nutrients [105]. These factors interact with the chemical and physical properties of soil, affecting the availability of essential macro- and micronutrients such as nitrogen (N), phosphorus (P), and potassium (K), along with secondary nutrients like calcium (Ca), magnesium (Mg), and sulfur (S) [106]. Soil texture plays a crucial role in nutrient retention, with sandy soils tending to have low nutrient-holding capacity, while clayey soils are more effective at retaining nutrients, though they may also pose challenges related to nutrient leaching or fixation [107]. Soils with high organic matter content demonstrate enhanced nutrient retention capabilities, primarily due to the formation of humus—a complex organic compound derived from decomposed organic matter. This humus acts as a crucial nutrient reservoir, absorbing essential elements and facilitating their gradual release over time, thereby significantly improving nutrient accessibility and availability to plant root systems [108].

Additionally, soil pH significantly affects the solubility and availability of nutrients. For instance, highly acidic soils may limit the availability of phosphorus, while alkaline soils can render micronutrients like iron and zinc less accessible to plants [109]. Moisture content and microbial activity also play a pivotal role in nutrient mineralization and cycling. Optimal moisture levels support microbial decomposition of organic matter, releasing plant-available forms of nitrogen, phosphorus, and sulfur. These interconnected processes influence the overall nutrient dynamics in open fields, necessitating continuous monitoring to ensure optimal soil health and fertility [110,111].

However, nutrients are not evenly distributed across fields, resulting in significant spatial variability. This variability is driven by factors such as topography, soil type, previous crop management practices, and natural nutrient cycling processes. For instance, sloped areas may experience greater erosion and nutrient loss, while low-lying areas with poor drainage may accumulate nutrients from water runoff, potentially leading to nutrient depletion over time [112]. Topography plays a key role, with higher elevations often suffering from nutrient leaching, whereas lower elevations may become nutrient-saturated, both of which require distinct management approaches [113].

Previous crop management practices, including fertilizer application and tillage, can create nutrient hotspots or deficiencies. Areas that have been over-fertilized in the past may develop toxicity, while others may remain deficient due to under-application or nutrient leaching [114]. Temporal variability in nutrient availability further complicates this, as factors such as seasonal changes, weather patterns, and crop growth stages all influence the nutrient status of the soil. During periods of rapid crop growth, plants can quickly deplete available nutrients, necessitating timely fertilization to maintain optimal growth conditions [115].

Given these challenges, precise nutrient sensing technologies are essential for capturing real-time data on nutrient status across different field areas. Traditional soil testing methods, which rely on random sampling and laboratory analysis, may not fully capture the spatial and temporal variability of nutrients. In contrast, advanced sensing tools, such as near-infrared spectroscopy (NIR), ion-selective electrodes (ISEs), and remote sensing technologies, provide more granular insights into nutrient dynamics [116,117]. This enables the development of site-specific management strategies, such as variable-rate fertilization (VRF), where nutrients are applied only where needed, and in the exact quantities required [118].

#### 3.2.2. Sensing Technologies for Soil Nutrients

Accurate and efficient sensing technologies are crucial for understanding and managing soil nutrient dynamics in open field soil cultivation. Traditional methods of soil nutrient assessment, which typically involve soil sampling followed by laboratory analysis, are time-consuming, costly, and often fail to capture the full extent of spatial and temporal nutrient variability within a field. The advancement of precision agriculture has driven the demand for real-time sensing technologies, offering high-resolution insights into soil nutrients for precise and efficient management [72,119].

**Figure 4:**
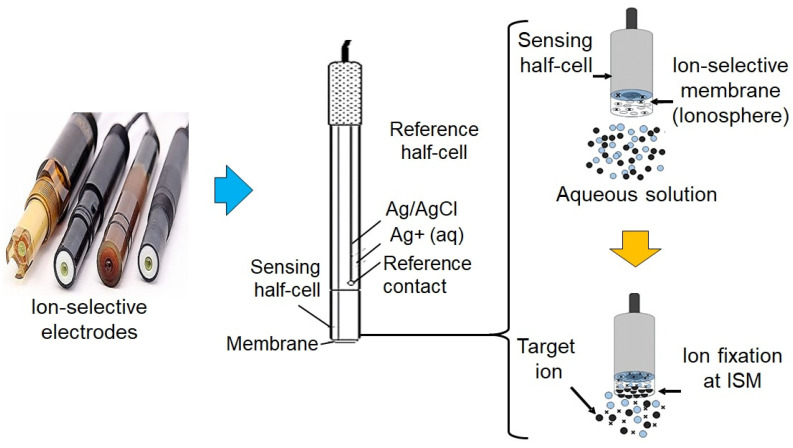
A schematic representation of the structure and function of ISEs for target ion detection in aqueous solutions is provided, illustrating their key operational principles and applications. This representation synthesizes insights obtained from a comparative analysis of soil properties, nutrient parameters, and advanced sensing techniques, as detailed in [122].

One of the most promising technologies in this space is near-infrared (NIR) spectroscopy uses light to measure the absorbance and reflectance of soil across different wavelengths, providing a rapid assessment of nutrient concentrations without the need for extensive soil preparation or laboratory analysis [120]. This technology is particularly effective for detecting organic matter, nitrogen, and moisture levels in soils. Its ability to offer real-time feedback makes it an invaluable tool for guiding precision nutrient management strategies, ensuring that nutrients are applied where they are needed most [121]. Another emerging technology is ion-selective electrodes (ISEs), which are capable of measuring the concentration of specific ions, such as nitrate (NO_3_^−^), potassium (K^+^), and phosphate (PO_4_^3−^), directly in the soil solution [122]. ISEs offer the advantage of being relatively low-cost and portable, making them well-suited for on-site, real-time monitoring of nutrient availability. Their specificity and sensitivity to particular nutrients enable farmers to quickly detect deficiencies or excesses in the soil, facilitating the development of tailored fertilization plans. Figure 4 shows a schematics of ISEs sensing technique.

**Figure 5:**
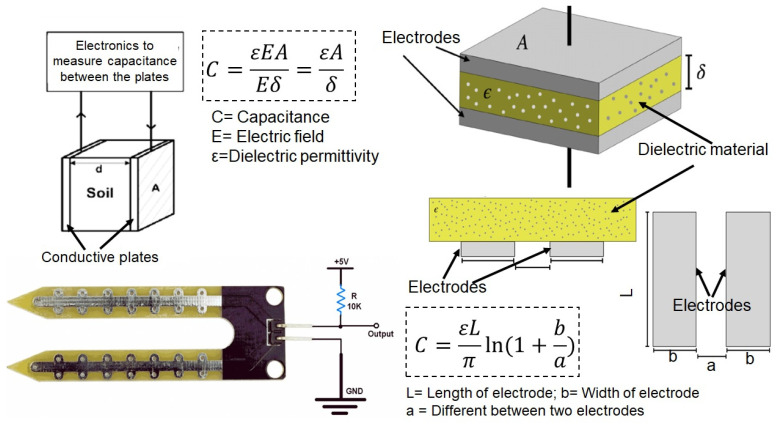
Schematic representation of the structure and measurement principles of a capacitance-based soil moisture sensor, showing key components such as conductive plates, dielectric material, and electrical circuit elements. This design estimates the changes in the dielectric constant to estimate moisture content, as simplified from in [123].

Electrochemical sensors are also gaining attention for their ability to monitor soil nutrients in situ [123]. These sensors can detect changes in the electrical properties of the soil as a response to the presence of specific nutrients, providing continuous data on nutrient concentrations [75,124]. Figure 5 illustrates a capacitance-based soil moisture sensor, which measures soil moisture by detecting changes in capacitance between two conductive plates. Capacitance changes as the dielectric constant of the soil varies, allowing the sensor to estimate water content. This real-time monitoring capability allows for more dynamic management of soil fertility, as nutrient levels can be adjusted throughout the growing season based on sensor feedback [72].

In addition to these ground-based sensors, remote sensing technologies are increasingly being used to assess soil nutrient status from an aerial perspective, offering large-scale insights into soil characteristics. Unmanned aerial vehicles (UAVs) with multispectral and hyperspectral cameras effectively monitor soil properties like organic matter, moisture, texture, and mineral composition, providing essential data linked to nutrient availability for precision agriculture [125].

Several vegetation indices, originally developed for plant analysis, have been adapted to indirectly estimate soil properties and nutrient levels [126,127]. The Soil-Adjusted Vegetation Index (SAVI) and the Transformed Soil-Adjusted Vegetation Index (TSAVI) are particularly effective for reducing the influence of soil background in areas with sparse vegetation [126]. These indices enhance soil signals, making them useful for monitoring soil conditions during early crop growth stages or in bare fields. Similarly, the Normalized Difference Soil Index (NDSI) highlights soil surface conditions such as crusting, compaction, and erosion, which can influence nutrient distribution [127]. By applying these indices, UAVs can detect spatial variations in soil properties that indicate nutrient deficiencies or imbalances [127].

Thermal infrared (TIR) imaging is another important tool in remote sensing, enabling the measurement of soil temperature [38]. Soil temperature plays a significant role in nutrient cycling processes, such as nitrogen mineralization, which directly impacts nutrient availability. Integrating TIR data with multispectral and hyperspectral imaging enhances the ability to identify field zones where temperature variations may affect nutrient dynamics [128]. For instance, while TIR imaging highlights temperature variations across a field, multispectral and hyperspectral imaging captures data on soil properties such as organic matter, moisture, and nutrient content [129]. When combined, these technologies can pinpoint specific zones within a field where temperature fluctuations may be influencing nutrient dynamics [38]. For example, cooler zones might indicate areas with higher soil moisture, which can slow down nitrogen mineralization, while warmer zones may correspond to drier soils, potentially affecting nutrient availability [129].

These technologies can then be combined with in-situ soil sampling and sensor data to create high-resolution nutrient maps, enabling farmers to make precise, site-specific nutrient management decisions. Remote sensing technologies can be combined with in-situ soil sampling and ground-based sensor data to generate high-resolution nutrient maps. UAVs equipped with advanced imaging systems can identify areas with optimal organic matter and moisture levels, indicating regions of higher nutrient availability. Conversely, zones with compacted or eroded soils can be identified, allowing targeted nutrient applications to improve fertility, providing farmers with actionable data for precise, site-specific management that enhances crop productivity while minimizing resource use [130]. Table 2 provides a concise overview of the major methods, offering a comparison of their applications, strengths, and limitations in soil nutrient sensing and precision agriculture.

#### 3.2.3. Sensor Platforms and Deployment

The effectiveness of soil nutrient sensing technologies largely depends on the platforms used for their deployment [28]. These platforms range from stationary ground-based systems to mobile and aerial units, each offering unique advantages and challenges based on the specific requirements of soil nutrient monitoring in open-field agriculture [25,28]. The choice of platform not only determines the scale and resolution of nutrient data collection but also influences the practicality of integrating the data into decision-making processes [28,118]. To optimize the benefits of nutrient sensing technologies, it is crucial to match the sensor platform with the appropriate deployment strategy for precise, site-specific nutrient management.

Ground-based sensor platforms provide localized, high-resolution data, often used for continuous monitoring of soil conditions in specific areas of a field. These platforms can be either stationary, with sensors permanently installed in the soil, or mobile, where sensors are mounted on tractors, or other agricultural machinery for flexible, on-the-go monitoring. Stationary platforms, such as soil probes equipped with ion-selective electrodes (ISEs) or electrochemical sensors, allow for constant monitoring of nutrient levels at fixed locations [122]. This approach is particularly valuable for tracking temporal variations in nutrient availability and identifying trends over time. However, the fixed nature of these sensors can limit their ability to capture the spatial variability of nutrients across larger fields. Ground-based “on-the-go” sensors have been developed to rapidly assess soil organic matter content, electrical conductivity, nitrate content, and compaction, and this data can complement image-based data to provide a more comprehensive understanding of soil properties across a field. Integrating ground-based sensor data with image-based data holds promise for enhancing soil property mapping and addressing spatial variability in crop production [12].

Aerial sensor platforms, particularly unmanned aerial vehicles (UAVs), have revolutionized the monitoring of soil nutrients by offering large-scale, high-resolution imaging capabilities. UAVs equipped with multispectral and hyperspectral sensors can capture detailed images of fields, which are then processed to produce maps of nutrient variability [131]. These maps provide farmers with an overview of nutrient distribution across vast areas, enabling them to identify nutrient hotspots or deficiencies and implement targeted management strategies [131]. Aerial platforms are particularly advantageous for monitoring difficult-to-reach areas, such as fields with uneven terrain or large expanses where ground-based monitoring would be impractical.

**Figure 6:**
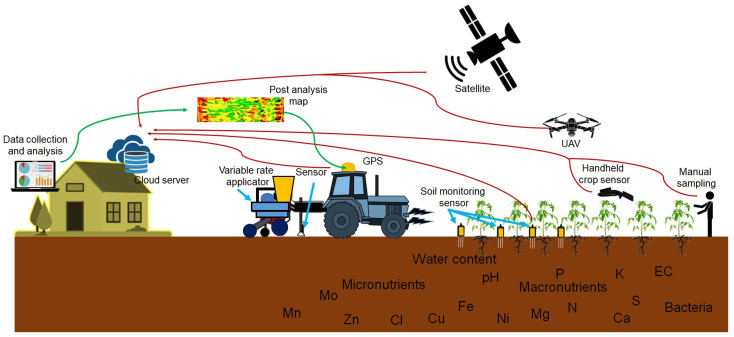
Integrated remote sensing and data collection platforms for soil nutrient and crop health monitoring in precision agriculture (modified from [132]).

Satellite-based sensor platforms also play a significant role in large-scale nutrient monitoring, especially in extensive agricultural regions. Although satellite data may not provide the same level of spatial resolution as UAVs or ground-based sensors, they offer the advantage of continuous, wide-area coverage. This makes satellites particularly useful for monitoring regional nutrient trends over time and assessing the impact of environmental factors, such as drought or excessive rainfall, on soil nutrient dynamics. Satellite imagery, when combined with ground-based measurements, can provide valuable insights into nutrient fluxes and inform decisions on nutrient replenishment across seasons [132]. Figure 6 illustrates a multi-platform approach to monitoring soil nutrient status and crop health in precision agriculture. It includes various technologies such as satellites, unmanned aerial vehicles (UAVs), GPS-equipped tractors, soil monitoring sensors, and handheld crop sensors.

In addition to the primary platforms for soil nutrient sensing, recent innovations in wireless sensor networks (WSNs) have enabled the development of distributed sensing systems that can cover an entire field [76,133]. WSNs consist of multiple low-cost, wireless sensors strategically placed throughout the field to monitor nutrient levels at different locations. These sensors communicate wirelessly with a central hub, which collects and processes the data in real time. The distributed nature of WSNs allows for continuous, field-wide monitoring without the need for manual intervention, significantly reducing labor costs and increasing the accuracy of nutrient assessments. The application of WSNs for continuous monitoring improved nutrient management by 30% compared to traditional manual sampling methods, highlighting the importance of real-time data for precision agriculture applications [134].

The deployment of sensor platforms must consider both the scale of the monitoring area and the specific nutrient management objectives. For small to medium-sized fields, ground-based platforms, whether stationary or mobile, may offer sufficient resolution and flexibility to manage nutrient variability. However, for larger fields or areas with significant spatial variability, fusion of ground-based sensing and aerial platforms such as UAVs or satellite imagery can provide a more comprehensive view of soil nutrient dynamics [135]. Moreover, the use of wireless sensor networks and advanced data processing algorithms can enhance the accuracy and timeliness of nutrient sensing, enabling farmers to make informed decisions on nutrient management in real-time [132]. Integrating ground-based, aerial, and wireless sensors provides a comprehensive understanding of soil nutrient variability, enabling precise and efficient management [134].

#### 3.2.4. Integration with Decision Support Systems

The integration of soil nutrient sensing technologies with Decision Support Systems (DSS) is a critical step toward effective, data-driven nutrient management in precision agriculture. DSS are computational tools that help farmers make informed decisions based on real-time data from various sensor platforms. By consolidating and analyzing data from ground-based, aerial, and satellite sensors, DSS can generate actionable insights that improve nutrient management, optimize resource use, and enhance crop productivity [136]. The seamless integration of nutrient sensing data into these systems allows for site-specific recommendations, contributing to both economic and environmental sustainability in open-field farming [25].

At the core of this integration is the ability of DSS to process large volumes of spatial and temporal data, turning raw sensor readings into practical information [137]. Soil nutrient levels, moisture content, crop health, and environmental conditions are analyzed together, enabling farmers to respond promptly to nutrient deficiencies or excesses [138]. For example, a DSS can analyze multispectral UAV data to detect nutrient stress in crops and recommend a targeted fertilization plan, ensuring that nutrients are applied only where needed, reducing wastage and the risk of runoff. The integration process begins with data acquisition, where sensing technologies provide real-time information on soil and crop conditions. This data is then fed into the DSS, which uses predictive models and machine learning algorithms to interpret the data and provide recommendations [139]. For example, soil nutrient readings from ion-selective electrodes (ISEs) deployed across a field can be uploaded to the DSS, which will assess spatial variability and suggest variable-rate fertilization (VRF) strategies [140]. By leveraging predictive analytics, the DSS can also forecast nutrient requirements based on crop growth stages, weather patterns, and historical nutrient data, helping farmers make proactive decisions [138].

One of the major benefits of integrating sensor data with DSS is the ability to implement precision agriculture practices such as VRF [118,141]. DSS can analyze soil nutrient maps and recommend precise amounts of fertilizer for different field zones. These recommendations are based on factors such as soil texture, nutrient levels, and moisture availability, ensuring that each area of the field receives the correct nutrient dosage [142]. This targeted approach not only maximizes crop yield but also minimizes the environmental impact of over-fertilization, which can lead to issues such as nutrient leaching and water contamination. Another key aspect of DSS integration is real-time monitoring and decision-making. Many modern DSS platforms are equipped with wireless communication capabilities, allowing sensor data to be transmitted instantly to the system. This enables farmers to monitor nutrient dynamics in real time and adjust their management practices accordingly [143]. For example, if soil moisture levels drop below a critical threshold, the DSS can trigger an irrigation event to maintain optimal conditions for nutrient uptake [142]. Similarly, if nutrient levels fall outside the desired range, the DSS can recommend immediate corrective actions, such as applying a specific fertilizer blend to restore balance [141].

The success of DSS integration depends not only on the accuracy of the sensor data but also on the system’s ability to present the information in a user-friendly manner. Modern DSS platforms often feature intuitive interfaces that allow farmers to visualize nutrient variability through maps, graphs, and alerts. This ease of access to information empowers farmers to make quick, informed decisions, without needing extensive technical expertise [144]. Additionally, many DSS platforms can be integrated with mobile applications, enabling farmers to receive recommendations and alerts directly on their smartphones, increasing the accessibility and practicality of precision nutrient management [145].

The integration of soil nutrient sensing technologies with DSS is a powerful tool for optimizing nutrient management in open-field farming. By combining real-time data with predictive analytics and user-friendly interfaces, DSS enables farmers to make data-driven decisions that enhance crop productivity, reduce input costs, and minimize environmental impacts. This integration is a foundation of precision agriculture, facilitating more sustainable and efficient farming practices that are responsive to the dynamic nature of soil nutrient dynamics [146].

### 3.3. Solution Nutrient Sensing for Facilitated Hydroponic Horticulture

Nutrient sensing within hydroponic solutions serves as an essential facet of precision horticulture, enabling growers to enhance plant development and optimize resource utilization. Cutting-edge technologies, such as ion-selective electrodes (ISEs), laser-induced breakdown spectroscopy (LIBS), and various other spectroscopic techniques, are fundamentally changing the landscape of nutrient monitoring through real-time, on-site assessments of critical elements like nitrogen, phosphorus, potassium, calcium, and magnesium [147].

The innovative systems allow for continuous tracking of nutrient levels, delivering accurate data that aids in making informed decisions about nutrient supplementation. By adopting these advanced monitoring solutions, hydroponic solution cultivation can significantly decrease water consumption, boost crop yields, and maintain a precise nutrient balance throughout the cultivation cycle. The technologies can identify minor fluctuations in nutrient concentrations early on, thereby enabling growers to promptly correct deficiencies or imbalances. This capacity is vital for ensuring the health and productivity of crops within soilless cultivation systems [148].

#### 3.3.1. Nutrient Management in Hydroponic Solution Cultivation Systems

**Figure 7:**
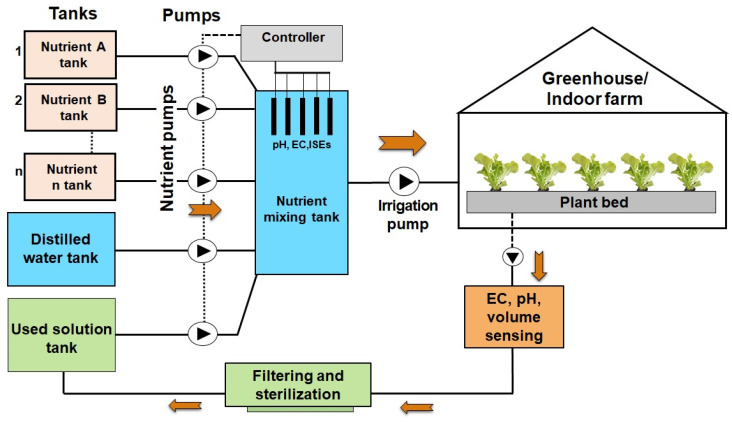
Schematic diagram of a general hydroponic solution nutrient management system, illustrating the key components and processes involved in maintaining optimal nutrient levels for plant growth. This diagram highlights the integration of sensors such as pH and EC sensors, automated nutrient dosing systems, and real-time monitoring platforms. It demonstrates the flow of nutrient solutions through recirculating systems, enabling precise control over macronutrients and micronutrients (adapted from [149,150]).

The implementation of effective nutrient management is crucial for enhancing crop productivity and sustainability within hydroponic solution cultivation systems, where plants rely entirely on nutrient solutions instead of soil to obtain essential minerals [149]. This soilless cultivation method offers the significant benefit of formulating nutrient solutions that are specifically tailored to the requirements of individual crops and their distinct growth phases. Such precision enables highly controlled delivery of macronutrients, including nitrogen, phosphorus, potassium, calcium, and magnesium, alongside vital micronutrients, thereby optimizing nutrient use efficiency and promoting desirable growth outcomes [150]. Figure 7 presents a schematic overview of a common hydroponic solution nutrient management system, designed to monitor and control nutrient levels for optimal plant growth.

**Figure 8:**
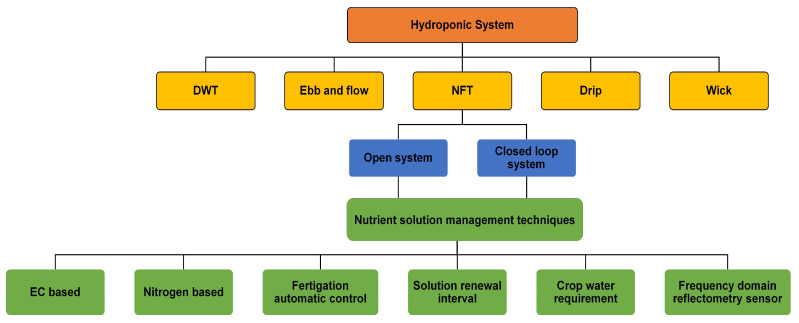
Hydroponic solution cultivation systems and nutrient solution management techniques, illustrating key methodologies. DWC: deep water culture; NFT: nutrient film technique. These are categorized into open and closed loop systems for nutrient solution management with different advanced nutrient management techniques, and automatic control. Adopted using frameworks and theoretical approaches outlined in [150].

In contrast to traditional soil-based agriculture, characterized by inconsistent nutrient availability, hydroponic solution cultivation systems provide nutrients in consistent and carefully regulated concentrations, facilitating efficient absorption [151]. Various nutrient delivery methods, such as the nutrient film technique (NFT), deep water culture (DWC), drip irrigation, and aeroponics, enhance nutrient availability at the root zone, thereby improving both growth rates and crop quality as shown in Figure 8.

Advanced monitoring technologies are integral to the successful management of hydroponic solution nutrients. For instance, laser-induced breakdown spectroscopy (LIBS) allows for precise, real-time quantification of nutrient concentrations, achieving relative errors as low as 6–13%, which facilitates prompt nutrient adjustments in alignment with current conditions. When combined with pH and electrical conductivity (EC) sensors, these systems maintain nutrient solutions within optimal pH ranges (5.5–6.5) and EC levels, both of which are critical for maximizing nutrient uptake [147]. These automated monitoring frameworks continuously modulate nutrient delivery to sustain an ideal nutrient equilibrium, thereby mitigating variability and minimizing the potential for deficiencies [152].

Recent innovations, notably Quantitative Nutrient Management (QNM), have demonstrated the capacity to decrease nutrient waste by approximately 60%, yielding both economic and environmental advantages without detracting from crop yield or quality. Furthermore, the advent of automated dosing systems and Internet of Things (IoT) integrated sensor networks facilitates real-time nutrient adjustments, significantly reducing fertigation volumes—by as much as 57.4%—while simultaneously enhancing crop growth outcomes compared to more conventional nutrient application practices [151].

Additionally, ongoing research into nutrient recovery from wastewater represents a critical advancement in promoting sustainability by enabling nutrient recycling. Treated wastewater has been shown to potentially fulfill up to 56% of hydroponic nutrient requirements [153]. These breakthroughs in nutrient recovery, paired with enhancements in sensor calibration and data processing, exemplify the next generation of hydroponic solution nutrient management, solidifying its significance in sustainable agriculture [51]. Such advanced, data-driven nutrient management techniques not only support resource conservation but also contribute to the reliable production of high-quality food [148].

#### 3.3.2. Key Sensing Technologies for Hydroponic Solution Cultivaiton

**Figure 9:**
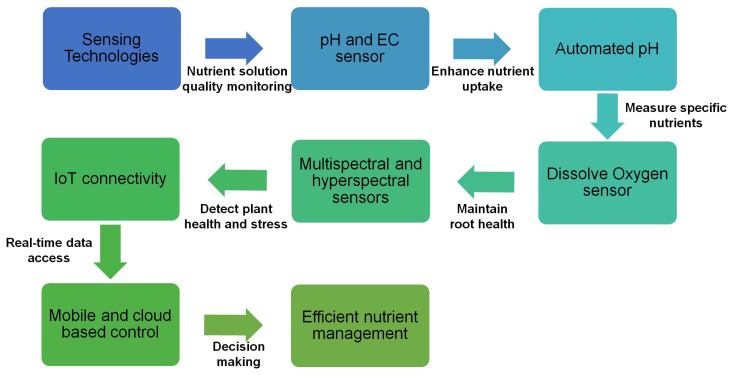
Flow diagram for nutrient sensing in hydroponic solution cultivaiotn in horticulture.

Sensing technologies play a crucial role in optimizing nutrient management within hydroponic solution cultivaiton systems by facilitating the precise delivery of essential nutrients necessary for optimal plant growth [44,46,147]. Figure 9 shows the flow diagram that illustrates the integration of various sensor-based technologies for nutrient sensing and management in hydroponic horticulture. Among the most important instruments are pH and electrical conductivity (EC) sensors, which enable real-time monitoring of the quality of nutrient solutions. Maintaining pH levels within the optimal range of 5.5 to 6.5 is critical for ensuring nutrient availability, as deviations can lead to nutrient deficiencies or toxicities that negatively impact plant health and yield [154]. Automated systems equipped with these sensors can dynamically regulate pH levels, thus enhancing nutrient uptake efficiency and improving overall crop performance.

Nutrient concentration sensors, which include ion-selective electrodes and optical sensors, have become increasingly important due to their capability to measure specific nutrient ions such as nitrate, potassium, and calcium in real-time. For example, research conducted by Kashyap and Kumar [83] indicated that the integration of ion-selective sensors into hydroponic solution cultivaiotn systems resulted in a 25% decrease in nutrient wastage and a substantial increase in crop yield.

Emerging technologies, such as hyperspectral imaging, provide detailed information regarding plant health, enabling the early detection of nutrient deficiencies and physiological stress. This capability is vital for timely interventions that can mitigate crop loss and boost productivity [155]. Moreover, the incorporation of these advanced sensing technologies with Internet of Things (IoT) connectivity allows for real-time data access and remote monitoring, empowering growers to make informed and swift decisions concerning nutrient delivery [56,139]. The conjunction with advanced nutrient sensors listed in Table 3, such as AI-based monitoring systems and automated moisture and nutrient sensors, these technologies collectively enable real-time data collection and analysis.

As these sensing technologies evolve, they hold the potential to significantly enhance nutrient management in hydroponic solution cultivaiton, fostering more efficient and productive cultivation practices. The continuous advancement and integration of sophisticated sensors, alongside data analytics, will not only improve resource-use efficiency but also contribute to sustainable agricultural practices and food security amid an increasingly challenging environmental landscape [160,161,162,163].

#### 3.3.3. Automation and Real-Time Monitoring

Automation and real-time monitoring are critical components of hydroponic solution cultivation, significantly enhancing the management of environmental conditions and nutrient delivery systems. Continuous supervision of essential parameters—such as humidity, temperature, water level, pH, and electrical conductivity (EC)—is fundamental to producing healthy, high-quality crops [21,40,44].

Different automated control and management system has been proposed specifically for tropical hydroponic solution cultivaiotn systems, which minimizes data exchange through multisensor fusion [165,166]. This approach intelligently aggregates sensor data, providing growers with accurate and timely information. For instance, Chaiwongsai [165] developed an automated sensing system employing ion-selective sensors to monitor nitrogen levels in open-field conditions. This system facilitated precise nutrient adjustments based on real-time feedback, achieving a detection accuracy of ±0.02 mg/L and requiring calibration only biweekly. Nitrogen use efficiency improved by 20%, while targeted nitrogen applications resulted in a 15% increase in crop yields for both wheat and corn. In hydroponic solution cultivaiton systems, pH and EC sensors are indispensable for monitoring nutrient solutions. Richa et al. [166] implemented automated control measures that maintained pH levels within the optimal range of 5.5 to 6.5 and EC levels between 1.8 and 2.2 mS/cm during lettuce cultivation. This real-time monitoring not only reduced water and nutrient wastage by 30% but also improved crop uniformity by 25%, yielding a 20% increase in overall crop growth rates compared to conventional manual management practices.

**Figure 10:**
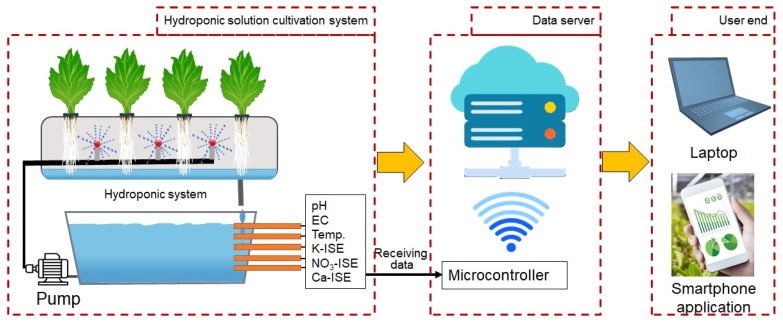
The system architecture of IoT-based hydroponics solution cultivaiton, highlighting the interconnection between various components, including sensors, microcontrollers, data servers, and user-end devices for real-time monitoring and automated nutrient management (modified and regenated from [168]).

The integration of Internet of Things (IoT) enabled gateways in hydroponic solution cultivation systems facilitates real-time data transmission and analysis. Recent studies by Shukla et al. [167] have shown that such systems can reduce data transmission latency to less than five seconds, enabling swift operator responses to nutrient imbalances. Smart sensing devices in hydroponics enable real-time monitoring of crop requirements, enhancing year-round production [168]. The automatic control system is capable of adjusting nutrient levels through an Android mobile application, ensuring that conditions remain optimal for plant growth as shown in Figure 10.

Furthermore, the capacity to detect power outages is critical in preventing disruptions to essential operations, such as nutrient solution pumping, thereby safeguarding plant health [168]. The integration of such advancements underscores the significant role of automation and real-time monitoring in fostering sustainable practices within tropical agriculture, effectively enhancing productivity while mitigating environmental impacts associated with hydroponic solution cultivation [147,167,168].

#### 3.3.4. Sensor Integration in Hydroponic Solution Cultivaiotn Systems

The incorporation of sophisticated sensors and intelligent algorithms into hydroponic solution cultivation systems has significantly transformed nutrient and environmental management, providing precise and adaptive control over vital parameters such as pH, electrical conductivity (EC), dissolved oxygen, and temperature [44,50]. These factors are crucial for optimizing crop growth and maximizing yield. Through real-time sensor fusion, hydroponic solution cultivation systems can achieve exceptional accuracy in monitoring and adjusting these variables, thereby greatly reducing reliance on manual intervention [136].

A sensor-fusion system developed by Rathor et al. [169] illustrates this innovative approach by establishing synchronized communication among sensors linked to a centralized server. This configuration allows for automated and intelligent decision-making based on environmental conditions. Utilizing a Random Forest algorithm, the system processes real-time data from sensors to identify and regulate the most critical parameters, effectively mitigating issues related to sensor crowding and optimizing resource utilization. Bhargava et al. [170] prioritizing temperature and water levels resulted in a 20.4% decrease in peak power consumption, while automatic adjustments to light levels achieved an outstanding 82.1% energy savings during peak times, thereby marking this model as an exceptionally energy-efficient solution for hydroponic solution operations.

Furthermore, certain sensors have proven essential in enhancing crop conditions. Massa et al. [171] emphasized the advantages of incorporating pH and EC sensors within a recirculating hydroponic solution setup, which enabled automated nutrient adjustments. This integration yielded a 25% reduction in nutrient consumption and a 20% increase in yields for leafy greens compared to conventional systems. By keeping nutrient levels within optimal ranges, this method bolsters plant health and diminishes the risks associated with nutrient toxicity or deficiency. Table 4 provides an overview of the primary accomplishments of sensor-based strategies aimed at minimizing water and nutrient losses in soilless vegetable cultivation, showing the diverse applications of sensor technologies in hydroponic solution cultivation.

The use of dissolved oxygen (DO) sensors is equally critical, maintaining levels above 6 ppm, which enhances root health, diminishes root disease incidence by 15%, and boosts biomass by 18% in leafy greens and herbs [179]. The implementation of IoT-enabled sensors facilitates remote monitoring and control through cloud-based dashboards, empowering operators to evaluate nutrient levels and environmental parameters in real-time [175,176]. This connectivity streamlines labor requirements and speeds up reactions to potential nutrient imbalances, ensuring consistently optimal conditions for plant growth [177]. Advancements in sensor fusion and intelligent algorithms underscore the potential for hydroponic solution cultivation systems to realize highly efficient, resource-conscious cultivation [34,178,179].

## 4. Discussion

Soil nutrient sensing technologies in modern agricultural practices offer significant potential for enhancing crop productivity and sustainability. However, several challenges and considerations must be addressed to ensure the effective deployment and utilization of these technologies. From technical limitations to environmental variability, the chal-lenges outlined below underscore the need for continuous refinement of nutrient sensing systems, calibration methods, and interpretation models. Understanding these hurdles will enable researchers and practitioners to mitigate risks, improve accuracy, and enhance decision-making in site-specific nutrient management.

One of the foremost challenges in soil nutrient sensing lies in the accuracy and relia-bility of sensor readings. Sensors used for soil nutrient analysis are highly sensitive to factors such as soil moisture, temperature, and texture [54]. Variability in these environ-mental factors can lead to inconsistencies in data, which can affect the precision of nutri-ent recommendations [78,180]. For example, sensors deployed in soils with high clay content may experience signal attenuation, while sandy soils may cause faster nutrient leaching, making it difficult to capture accurate readings over time [134]. Moreover, fluctuating moisture levels can cause rapid changes in nutrient availability, complicating the inter-pretation of sensor data and necessitating frequent recalibration of the sensing systems.

Another key challenge is the spatial heterogeneity of soil nutrients across open field soil cultivation. Although advanced sensing technologies provide granular data, the spa-tial variability of nutrients, driven by differences in topography, soil type, and manage-ment practices, requires robust interpolation techniques to generate accurate nutrient maps [181]. In some cases, even the most sophisticated sensors may not fully capture the complex spatial distribution of nutrients. This raises the need for combining multiple data sources, such as remote sensing, ground-based sensors, and traditional soil sampling, to build a comprehensive picture of soil nutrient dynamics.

Cost and accessibility are additional considerations when deploying soil nutrient sensing systems, especially for small-scale farmers [83]. Advanced sensors, UAVs, and related technologies can be prohibitively expensive, limiting their widespread adoption. Additionally, the costs associated with data processing, maintenance, and regular sensor calibration can place further financial strain on farmers. Reducing the cost of these tech-nologies through innovation, subsidies, or scaling production is crucial for making preci-sion agriculture more accessible to a broader range of agricultural operations [182].

Furthermore, integrating sensor platforms with decision support systems (DSS) brings its own set of challenges [183]. While DSS can provide valuable insights for nutri-ent management, the accuracy of the recommendations depends heavily on the quality and timeliness of the data input [146]. If the data fed into the system is outdated or inac-curate, the resulting decisions may lead to under- or over-application of fertilizers, causing either nutrient deficiencies or environmental harm due to runoff and leaching [184]. This challenge underscores the importance of real-time data collection, seamless integration, and continuous updates to DSS algorithms to ensure effective nutrient management. To ensure the long-term sustainability and effectiveness of soil nutrient sensing technologies, it is crucial to account for the environmental factors that can influence their accuracy and performance. Unlike plant nutrient sensing methods, such as multispectral imaging, which are affected by external conditions like weather and lighting [185], soil nutrient sensing technologies must be robust enough to provide reliable data across varying environmental scenarios. Careful consideration of these factors can enhance the precision and sustainability of nutrient management practices over time. While these systems are de-signed to optimize fertilizer use and reduce nutrient runoff, there is always a risk that im-proper use of the technology or incorrect sensor readings could exacerbate environmental issues, such as groundwater contamination or soil degradation. Therefore, establishing standard guidelines for the application of nutrient sensing technologies, including the ethical use of data and its implications for ecosystem health, is essential for promoting sustainable agriculture.

Nutrient sensing in hydroponic solution cultivation systems faces significant challenges but also offers exciting prospects for precision horticulture. While sensors for pH and electrical conductivity are widely available, accurately measuring specific nutrients like nitrogen, phosphorus, and potassium is difficult due to the complexity of nutrient solutions. Current sensors often struggle with sensitivity and selectivity, leading to inaccuracies that can affect plant health. Future advancements aim to improve nutrient sensing through ion-selective electrodes (ISEs) and optical sensors, which promise better sensitivity and quicker responses. Integrating AI for predictive analysis could allow for dynamic nutrient adjustments based on plant needs, potentially increasing yields by 20–30% and reducing waste by up to 25%.

Hydroponic cultivation offers a highly efficient method for nutrient management, particularly in resource-limited or controlled environments, but it cannot completely replace soil cultivation for all crops and contexts [186]. Hydroponic systems excel in optimizing water and nutrient use, enabling precise delivery to plants and supporting higher yields compared to conventional soil-based agriculture, especially for leafy greens and vegetables [187]. However, soil provides a natural medium rich in organic matter, microbiomes, and micronutrients that are challenging to replicate in hydroponics [177,178]. Hydroponics also demonstrates superior resource efficiency, particularly in water conservation and land use [188], while often exhibit faster growth and higher yields, soil-grown crops may have enhanced flavor profiles and better mineral diversity due to complex soil-plant interactions [189]. Furthermore, soil cultivation remains more feasible for large-scale staple crops like wheat, corn, and rice due to its lower dependency on infrastructure and energy inputs [190]. Thus, hydroponics complements but does not completely replace soil cultivation, with quality and feasibility varying depending on crop type and cultivation goals.

Soil nutrient dynamics in open field soil cultivation are subject to high variability due to natural factors such as rainfall, temperature changes, and biological activity. This temporal and spatial variability poses challenges for accurate nutrient monitoring. Many existing nutrient sensing technologies, such as laboratory-based methods, in-situ soil moisture sensing methods, and remote sensing approaches, have limitations in capturing the spatial and dynamic variability of soil properties [83]. In-situ methods provide only point-based measurements, failing to account for field-wide spatial variability [191], while remote sensing techniques, which assess factors like vegetation cover, soil moisture, and surface roughness [192], and proximal in-field monitoring systems are not effective in tracking dynamic shifts in nutrient levels across large areas [48]. Furthermore, traditional sensor networks like coarse sampling rate and dependence of external factors often struggle to offer continuous, high-resolution data due to the heterogeneity of soil, leading to inaccurate nutrient recommendations and inefficient use of fertilizers [193,194]. Real-time continuous soil monitoring (RTCSM) and soil management data collection in open fields is also complicated by varying heterogeneous soil parameters and diverse pollutants and other environmental conditions that impact sensor performance [136]. Current technologies often provide delayed or averaged data, which can result in crop nutrition deficiencies or surpluses, reducing overall productivity.

Current sensing technologies for hydroponic solution cultivation systems, such as electrochemical sensors for pH, remain a major obstacle to the large-scale adoption of precision agriculture because of their inability to provide comprehensive nutrient data [195] and too high or too low electrical conductivity (EC) measurements would induce nutrient stress and increase plant antioxidant enzyme activities, which are limited to pakchoi growth and quality [22,196]. While pH and electrical conductivity (EC) sensors are commonly used in hydroponics to monitor general nutrient availability [197]. Calcium electrodes are limited in providing less sensitivity in detection in hydrophonic solutions [166]. Current methods employed in most commercial farms for online nutrient supply monitoring are limited to pH and conductivity measurements. These techniques can only offer an indication of the overall change in the complex nutrient mixture and lack the capability to precisely identify the specific nutrient or quantify the nutrient content. Most of the existing techniques for measuring individual nutrient levels are expensive and invasive, necessitating sample preparation, frequent recalibration, and skilled personnel for operation [147].

Furthermore, in hydroponic solution cultivation production systems, accurate and precise sensing of ion-selective electrodes is essential for real-time ion-specific nutrition management. The pH of the fluid, temperature, motion state, and amount of nutrient mixing can all have an impact on sensing performance [198]. The EC and pH of hydroponic solutions are typically employed in greenhouses and plant factories to assess their quality. But EC and pH don’t tell us enough about ion imbalances in hydroponic solutions, which could lead to poor yields or nutrient waste [48].

Real-time monitoring of nutrient solutions in hydroponic solution cultivation faces several challenges, primarily due to environmental variability and the need for precise adjustments [199]. One of the main challenges in a greenhouse environment is to keep up with the environmental parameters like temperature, humidity, etc. at a specific level [200], another one is the dynamic nature of nutrient consumption by plants, which can vary based on environmental conditions, plant growth stages, and water uptake rates [201]. These fluctuations make it difficult for sensors and automation systems to maintain optimal nutrient levels in real-time. Additionally, automation systems in hydroponic solution cultivation are often programmed based on pre-set parameters, meaning they use predefined thresholds that assume relatively stable conditions. These fixed parameters lack the flexibility to respond quickly to the rapid changes in nutrient concentration or pH that can occur during different plant growth stages or in response to external environmental shifts such as temperature changes [202].

Another significant limitation is related to the performance of sensors used in hydroponic systems. Many hydroponic solution sensors, such as pH and EC sensors, are sensitive to temperature fluctuations, which can affect the accuracy of their readings [203]. These inaccuracies make it challenging to perform timely adjustments in nutrient dosing. Furthermore, the speed and resolution of certain sensor technologies may be insufficient for dynamic control. For example, TDS sensors and simple microcontroller-based systems often lack the ability to provide high-frequency measurements needed for immediate corrective actions, resulting in delays between data acquisition and system response [204].

To address the limitations in existing soil and nutrient sensing technologies for open-field and hydroponic systems, significant advancements are required in areas such as sensor design, data integration, and real-time response systems. For open-field systems, research should focus on developing hybrid sensors that combine electrochemical and optical sensing to capture dynamic soil nutrient changes with high spatial and temporal accuracy. Integration of multi-sensor data with machine learning algorithms can enhance predictions of nutrient dynamics, enabling precise site-specific management. Additionally, the development of robust, miniaturized sensors with self-calibration capabilities can improve resilience in heterogeneous soil environments. In hydroponic systems, the current reliance on pH and electrical conductivity (EC) sensors needs to be expanded with ion-specific sensors capable of detecting critical nutrients such as nitrogen and potassium with high sensitivity. These sensors should also incorporate advanced materials to resist environmental factors such as temperature fluctuations. Machine learning models can further optimize nutrient management by dynamically adjusting dosing systems based on real-time data and environmental conditions like humidity and temperature. High-frequency measurement systems and adaptive automation technologies are essential for providing continuous, actionable feedback to prevent nutrient imbalances. A methodological framework encompassing lab-based sensor development, field validation, and data-driven modeling will enable scalable, cost-effective solutions for both small-scale and commercial farming operations. These advancements will show the way for more precise, efficient, and sustainable nutrient management in modern agriculture. Table 5 provides an overview of the current challenges in soil and hydroponic nutrient sensing technologies, along with innovative approaches and future research directions for smart agriculture applications.

The manuscript bridges a critical gap by comparing nutrient sensing technologies in open-field and hydroponic systems, highlighting their differences and similarities. Open-field technologies, such as electrochemical sensors and remote sensing platforms, address challenges like spatial and temporal variability in heterogeneous soils. Conversely, hydroponic systems rely on precise monitoring tools, including ISEs and pH sensors, to maintain optimal nutrient balance in controlled environments. While open-field systems manage environmental variability like rainfall and erosion, hydroponics demand real-time feedback for dynamic adjustments. Previous reviews often focus on one domain, neglecting cross-applicability of technologies. This manuscript highlights shared challenges, such as sensor accuracy and cost, while proposing integration to enhance efficiency and sustainability.

The manuscript provides a holistic analysis of nutrient sensing technologies across both systems, synthesizing advancements such as hybrid sensors, IoT-enabled platforms, and AI applications. Unlike earlier works, it evaluates adaptability across diverse agricultural contexts, linking technologies with precision agriculture practices like real-time automation and DSS. These insights address critical challenges, reduce resource waste, and emphasize sustainable agricultural practices. By offering a comprehensive analysis of nutrient sensing across open-field and hydroponic systems, the manuscript addresses gaps in previous literature. It demonstrates how technologies like optical spectroscopy and AI-driven models can be applied across systems, ensuring site-specific nutrient management and advancing sustainability goals.

However, the manuscript has limitations. It does not extensively explore greenhouse cultivation, a hybrid system with unique challenges like microclimatic variability. Additionally, the use of a literature review limited the breadth and reproducibility of included studies. Future research should adopt systematic reviews to ensure comprehensive synthesis and include underexplored contexts, such as greenhouse systems and aquaponics. Bibliometric analysis can also reveal trends and address gaps in nutrient sensing technologies more systematically.

## 5. Conclusions

The increasing demand for sustainable agriculture highlights the essential role of nutrient sensing technologies in open-field and hydroponic systems. This review bridges a critical gap by comparing these technologies, highlighting their unique applications and cross-applicability. Open-field systems rely on tools like electrochemical sensors and remote sensing platforms to address challenges such as spatial and temporal variability in heterogeneous soils, while hydroponic systems demand precise, real-time monitoring using ion-selective electrodes (ISEs) and pH sensors to maintain nutrient balance in controlled environments. This comparison underscores shared challenges, such as sensor accuracy, calibration, and cost, while emphasizing opportunities for integrating advancements to enhance efficiency and sustainability.

The manuscript contributes significantly by synthesizing innovations like hybrid sensors, IoT-enabled platforms, and AI applications, evaluating their adaptability across diverse agricultural contexts. By linking these technologies with precision agriculture practices such as real-time automation and DSS, it provides actionable insights to improve resource efficiency and reduce environmental impact. Unlike earlier studies that focus exclusively on either open-field or hydroponic systems, this review offers a holistic perspective, addressing gaps in the literature and advancing the understanding of nutrient sensing technologies for site-specific management.

Despite its contributions, the manuscript has limitations. It does not extensively address greenhouse cultivation, a hybrid system that introduces unique challenges such as microclimatic variability and its impact on nutrient sensing. Additionally, the use of a narrative literature review limited the breadth and reproducibility of included studies, potentially overlooking critical advancements. These limitations highlight the need for future research to adopt systematic review methodologies, explore greenhouse systems and aquaponics, and incorporate bibliometric analyses to identify emerging trends and gaps systematically.

Future research should focus on developing affordable, multi-functional sensors that provide accurate real-time data on soil conditions. Integrating nutrient sensing technologies with predictive models and big data platforms will further enhance decision-making and optimize resource use. Additionally, addressing the unique challenges of open-field and hydroponic solution cultivation systems through targeted innovations will be essential for maximizing the potential of these technologies. By advancing soil nutrient sensing, agriculture can move toward more sustainable practices, ensuring higher productivity while minimizing environmental impacts.

## Figures and Tables

**Table 1:** Systematic categorization of reviewed documents based on core sub-sections in nutrient sensing technologies.

Section	Sub-Section	Documents Reviewed	Key Focus Area
Nutrient sensing	Key soil sensing parameters	35 *	Soil properties like pH, organic matter, moisture, and essential macronutrients (N, P, K)
	Soil nutrient sensing techniques	42 *	Advances in sensing technologies, including electrochemical sensors, optical sensors, and remote sensing
	Importance of soil nutrient parameters	40 *	Critical role of parameters in soil fertility, crop health, and sustainable management
Soil nutrient sensing for site-specific open field management	Soil nutrient dynamics in open fields	45 *	Variability in soil nutrients due to physical, chemical, and biological processes and implications for site-specific nutrient management.
	Sensing technologies for soil nutrients	50 *	Real-time sensing tools like NIR spectroscopy, ion-selective electrodes (ISEs), and remote sensing for soil nutrient assessment.
	Sensor platforms and deployment	30 *	Deployment of sensing systems on platforms such as UAVs, tractors, and stationary systems for dynamic field-level monitoring.
	Integration with decision support systems	25 *	Role of DSS in data-driven decision-making, variable-rate fertilization, and integration with real-time sensor data.
Solution nutrient sensing for facilitated hydroponic horticulture	Nutrient management in hydroponic solution cultivation systems	48 *	Tailored nutrient delivery in hydroponic systems to optimize macronutrient and micronutrient concentrations.
	Key sensing technologies for hydroponic solution cultivation	54 *	Advanced sensing tools like pH, EC, and ion-selective electrodes integrated with hydroponic nutrient management systems
	Automation and real-time monitoring	38 *	IoT-based systems and automated nutrient adjustments for real-time feedback and efficiency in hydroponic systems.
	Sensor integration in hydroponic solution cultivation systems	33 *	Integration of sensor data for optimized nutrient control using AI and predictive models in hydroponic cultivation.

* Each sub-section contains same/similar article in common.

**Table 2:** Overview of major methods in soil nutrient sensing with comparison of their applications for precision agriculture.

Method	Principle	Major Parameter	Advantages	Disadvantages
NIR spectroscopy	Measures absorbance and reflectance of light across different wavelengths	Organic matter, nitrogen, moisture	Non-invasive, real-time feedback, rapid nutrient assessment	Limited to surface-level analysis, less effective for certain soil types
Ion-selective electrodes (ISEs)	Detect specific ion concentrations based on selective ion exchange membranes	Nitrate (NO_3_^−^), Potassium (K^+^), Phosphate (PO_4_^3−^)	Low cost, portable, real-time ion-specific monitoring	Requires calibration, limited sensitivity for some ions, prone to fouling
Electrochemical sensors	Measures changes in electrical properties (e.g., conductivity, potential) in response to nutrient presence	General nutrient levels, soil salinity	Continuous monitoring, suitable for in-situ applications	Limited ion specificity, affected by soil heterogeneity and environmental factors
Capacitance-based soil moisture sensors	Measures changes in capacitance caused by soil’s dielectric constant variations	Soil moisture content	Simple design, cost-effective, real-time monitoring	Limited to moisture measurement, less effective in highly saline soils
Multispectral/hyperspectral imaging	Captures spectral data from aerial perspectives to assess soil properties	Organic matter, moisture, texture, mineral composition	Large-scale, non-invasive, suitable for sparse vegetation areas.	High initial cost, lower resolution compared to in-situ methods.
Vegetation indices (SAVI, TSAVI, NDSI)	Enhances soil and vegetation signals through spectral index calculations.	Soil crusting, compaction, erosion, nutrient distribution	Effective in sparse vegetation fields, monitors spatial variability.	Indirect measurements, less effective in densely vegetated areas.
Thermal infrared (TIR) imaging	Measures soil temperature variations using thermal radiation.	Soil temperature	Identifies temperature effects on nutrient cycling, complements spectral data.	Temperature data alone cannot quantify nutrient levels.
Integrated mapping (UAV + in-situ sensors)	Combines remote sensing and ground-based data for high-resolution mapping.	Multiple soil properties (nutrients, moisture, temperature)	High-resolution, field-specific nutrient maps, actionable insights for management.	High data integration complexity, requires expertise for interpretation.

**Table 3:** Summary of recent technological advancements in hydroponic solution cultivaiton.

Hydroponic Technology	Key Benifits	Primary Limitaitons	Reference
AI-driven monitoring	High accuracy in nutrient and pH management, yield maximization	Expensive, requires technical expertise	[156,157,158]
Precision farming	Optimized resource utilization, enhanced crop quality	High initial setup cost, complex to implement	[159,160]
Advanced sensors	Real-time data tracking, better water management	Installation and upkeep costs, risk of technical issues	[161]
Automated environmental control	Accurate climate regulation, better crop quality, and yield	High energy demand, operational expenses	[162,163]
Full-spectrum LED lighting	Energy-efficient, promotes plant growth	High setup cost, possible plant stress if not carefully managed	[162]
Mobile applications	Remote monitoring and control, user-friendly	Dependent on connectivity, app feature limitations	[164]

**Table 4:** An overview of sensor-based strategies for reducing water and nutrient losses in soilless vegetable cultivation.

Sensor-Based Approach	Sensors/Devices	Crops	Main Achievements	Reference
On-demand irrigation based on substrate water status sensing	Tensiometer	Tomato, Green Bean, Strawberry	Optimized nutrient solution scheduled to plant needs; savings ±20% water use efficiency over timer-based systems; low leaching fraction (<10%)	[172,173,174]
Dielectric soil moisture sensors	Dielectric moisture sensors	Tomato, Lettuce, Basil	Real-time substrate moisture monitoring to improve water application and prevent over-watering	[172,175]
Leachate volume and EC monitoring	Fertigation control tray	Tomato, Cucumber	Automatic fertigation based on LF and EC targets, achieving precise control with low-cost equipment	[176]
Plant stress sensing	Hyperspectral sensors	Tomato	Early detection of plant stress (water, nutrients, salinity) for proactive regulation of water and fertilization	[177]
Integrated sensors on the IoT platform	EC, pH, liquid counter, flow meter, climatic sensors	Tomato	Water consumption was reduced by 30% and fertilizer savings by up to 80% compared to empirical management	[178]

**Table 5:** Technical summary of different approaches, and future research directions for advancing nutrient sensing technologies in precision agriculture.

Aspect	Limitations	Approaches	Future Directions
Open-field soil sensing	Inability to capture field-wide spatial variability; reliance on point-based data; low resolution of traditional sensors.	Hybrid sensors combining electrochemical and optical technologies; multi-sensor data fusion.	Development of miniaturized, self-calibrating sensors; machine learning integration for site-specific nutrient management.
Hydroponic systems	Limited to pH and EC measurements; inability to detect specific ions; sensitivity to temperature fluctuations.	Ion-specific sensors for real-time monitoring; advanced materials for sensor durability.	Integration of adaptive automation systems with dynamic thresholds; high-frequency sensing technologies.
Data integration and analysis	Delayed or averaged data; insufficient data resolution for real-time decision-making.	Machine learning models for nutrient prediction; IoT-enabled real-time data collection.	Enhanced AI algorithms for predictive analytics; user-friendly platforms for data-driven decision-making.
Environmental variability	Impact of temperature, humidity, and pollutants on sensor performance.	Sensors with environmental resilience; multi-modal sensing systems.	Developing robust sensors that adapt to environmental changes; integrating environmental data into nutrient management systems.
Automation in agriculture	Pre-set thresholds limiting responsiveness to rapid changes; lack of flexibility in current systems.	Adaptive algorithms for real-time adjustments; IoT-based remote monitoring systems.	Scalable automation frameworks for small and large farms; real-time corrective feedback mechanisms.

## Data Availability

Data is contained within the article.
